# Biomechanical Assessment of Liver Integrity: Prospective Evaluation of Mechanical Versus Acoustic MR Elastography

**DOI:** 10.1002/jmri.29560

**Published:** 2024-08-21

**Authors:** Vitali Koch, Jennifer Gotta, Victoria Chernyak, Duygu Cengiz, Katerina Torgashov, Katrin Eichler, Valérie Vilgrain, Simon S. Martin, Nicole S. Ziegengeist, Paul Konrad, Christian Booz, Ibrahim Yel, Tommaso D'Angelo, Scherwin Mahmoudi, Jan‐Erik Scholtz, Simon Bernatz, Leona S. Alizadeh, Marina Cimprich, Levent A. Solim, Axel Thalhammer, Tatjana Gruber‐Rouh, Renate M. Hammerstingl, Stefan Zeuzem, Fabian Finkelmeier, Anita Pathil‐Warth, Melis Onay, Maximilian N. Kinzler, Omar Darwish, Giacomo Annio, Stuart A. Taylor, Peter Wild, Iulia Dahmer, Eva Herrmann, Haidara Almansour, Thomas J. Vogl, Leon D. Gruenewald, Ralph Sinkus

**Affiliations:** ^1^ Department of Diagnostic and Interventional Radiology Goethe University Frankfurt, University Hospital Frankfurt am Main Germany; ^2^ Department of Radiology Memorial Sloan Kettering Cancer Center New York City New York USA; ^3^ Laboratory of Imaging Biomarkers, Center for Research on Inflammation, UMR 1149 INSERM, Université de Paris Paris France; ^4^ Department of Radiology Beaujon University Hospital Paris Nord, AP‐HP Clichy France; ^5^ Department of Internal Medicine I Goethe University Frankfurt, University Hospital Frankfurt am Main Germany; ^6^ School of Biomedical Engineering and Imaging Sciences, King's College London London UK; ^7^ Laboratory of Translational Vascular Sciences, U1148, INSERM, Université de Paris Paris France; ^8^ Radiology Department University College London Hospitals NHS Foundation Trust London UK; ^9^ Dr. Senckenberg Institute of Pathology, Goethe University Frankfurt, University Hospital Frankfurt am Main Germany; ^10^ Institute of Biostatistics and Mathematical Modeling, Faculty of Medicine, Goethe University Frankfurt Frankfurt am Main Germany; ^11^ Department of Diagnostic and Interventional Radiology Eberhard‐Karls University Tuebingen Tuebingen Germany

**Keywords:** steatohepatitis, magnetic resonance elastography, magnetic resonance imaging, liver fibrosis, fatty liver

## Abstract

**Background:**

Magnetic resonance elastography (MRE) can quantify tissue biomechanics noninvasively, including pathological hepatic states like metabolic dysfunction‐associated steatohepatitis.

**Purpose:**

To compare the performance of 2D/3D‐MRE using the gravitational (GT) transducer concept with the current commercial acoustic (AC) solution utilizing a 2D‐MRE approach. Additionally, quality index markers (QIs) were proposed to identify image pixels with sufficient quality for reliably estimating tissue biomechanics.

**Study Type:**

Prospective.

**Population:**

One hundred seventy participants with suspected or confirmed liver disease (median age, 57 years [interquartile range (IQR), 46–65]; 66 females), and 11 healthy volunteers (median age, 31 years [IQR, 27–34]; 5 females).

**Field Strength/Sequence:**

Participants were scanned twice at 1.5 T and 60 Hz vibration frequency: first, using AC‐MRE (2D‐MRE, spin‐echo EPI sequence, 11 seconds breath‐hold), and second, using GT‐MRE (2D‐ and 3D‐MRE, gradient‐echo sequence, 14 seconds breath‐hold).

**Assessment:**

Image analysis was performed by four independent radiologists and one biomedical engineer. Additionally, superimposed analytic plane shear waves of known wavelength and attenuation at fixed shear modulus were used to propose pertinent QIs.

**Statistical Tests:**

Spearman's correlation coefficient (*r*) was applied to assess the correlation between modalities. Interreader reproducibility was evaluated using Bland–Altman bias and reproducibility coefficients. *P*‐values <0.05 were considered statistically significant.

**Results:**

Liver stiffness quantified via GT‐2D/3D correlated well with AC‐2D (*r* ≥ 0.89 [95% CI: 0.85–0.92]) and histopathological grading (*r* ≥ 0.84 [95% CI: 0.72–0.91]), demonstrating excellent agreement in Bland–Altman plots and between readers (*κ* ≥ 0.86 [95% CI: 0.81–0.91]). However, GT‐2D showed a bias in overestimating stiffness compared to GT‐3D. Proposed QIs enabled the identification of pixels deviating beyond 10% from true stiffness based on a combination of total wave amplitude, temporal sinusoidal nonlinearity, and wave signal‐to‐noise ratio for GT‐3D.

**Conclusion:**

GT‐MRE represents an alternative to AC‐MRE for noninvasive liver tissue characterization. Both GT‐2D and 3D approaches correlated strongly with the established commercial approach, offering advanced capabilities in abdominal imaging compared to AC‐MRE.

**Evidence Level:**

1

**Technical Efficacy:**

Stage 2

Following a drastic increase in incidence and prevalence over the past three decades, metabolic dysfunction‐associated steatotic liver disease (MASLD) has now become the primary cause of end‐stage liver disease worldwide.^1^ Ongoing societal and dietary changes are expected to intensify this development further, placing a growing strain on healthcare systems.^2^ Despite no to mild symptoms in the early stages, a gradual progression from simple steatosis to metabolic dysfunction‐associated steatohepatitis (MASH) with fibrosis and, ultimately, liver cirrhosis has been observed.^3^ The insidious disease progression underscores the significance of identifying and evaluating patients in early disease stages, in which lifestyle and dietary interventions are most effective.^4^ As new therapeutic strategies emerge, the need to noninvasively assess treatment efficacy and monitor the course of disease will gain even greater significance.^5^

Liver biopsy remains the reference standard for the grading of MASH. It is, however, currently not consistently performed for assessing MASH or MASLD due to its limitations, including bleeding complications, costs, and sampling errors.^6^, ^7^ Instead, a wide array of noninvasive tests is available in clinical practice, including laboratory‐based scoring systems and imaging‐based approaches like magnetic resonance elastography (MRE) or ultrasound elastography, often used in conjunction.^8^, ^9^, ^10^

MRE is established as the most accurate imaging method for identifying and staging liver fibrosis.^11^, ^12^, ^13^, ^14^, ^15^, ^16^, ^17^ Due to visualization and assessment of a large portion of the liver, MRE can capture focal heterogeneities and allows for a global assessment, in contrast to liver biopsy or ultrasound elastography. In commercially available acoustic (AC) transducers approved as Medical Device, shear waves of a constant frequency, typically at 60 Hz, are imaged using motion‐sensitive MR‐imaging sequences at several phases of the motion cycle.^18^, ^19^ AC drivers, however, are inherently nonlinear and suffer from silencing when too much preload is applied, resulting in a deterioration of the wave quality due to upper harmonics or reduced amplitude, respectively.^17^, ^20^

The gravitational (GT) transducer is a new technique based on an entirely different approach for creating shear waves when compared to the AC concept: it uses an eccentrically rotating mass for generating mechanical vibrations.^21^ This approach maintains a consistent vibration amplitude across various driving frequencies and delivers, due to its design, a vibrational amplitude independent from any preload condition. It thereby facilitates deeper organ penetration of shear waves over a wider frequency range free from relevant upper harmonic degradation. This capability may enhance the accuracy of wave maps, potentially improving the quality of viscoelastic reconstructions necessary for advanced tissue characterization. To date, GT‐MRE has only been tested in small groups of healthy volunteers and patients with liver conditions, offering only preliminary results.^21^, ^22^ The present study aims to compare the diagnostic performance of GT 2D/3D‐MRE using a gradient‐echo (GRE) acquisition with AC 2D‐MRE using a spin‐echo (SE) echo‐planar imaging (EPI)‐based acquisition in a large clinical cohort of patients with liver disease, fully embedded into routine clinical practice.

## Materials and Methods

The institutional ethical review board approved this prospective study. Written informed consent was obtained from all participants.

### MRE Protocol

MRE was performed on a 1.5 T MR scanner (MAGNETOM Aera, Siemens Healthineers, Erlangen, Germany), starting with the commercial AC 2D‐MRE protocol (Resoundant, Rochester, Minnesota, USA) and followed by 2D and 3D image acquisition protocols using the research GT transducer. The entire clinical MR protocol comprised anatomical fat‐saturated T1‐weighted 3D gradient‐echo Dixon VIBE (volumetric interpolated breath‐hold examination), T2‐weighted 2D turbo spin‐echo, and diffusion‐weighted 2D sequences as part of the department's routine liver protocol. The commercial MRE sequence employed was a motion‐sensitive 2D SE‐based sequence with echo‐planar readout (acquisition matrix 100 × 100 pixels; frequency 60 Hz; no. of slices 5; slice thickness 8 mm), while for GT MRE GRE sequences were used (acquisition matrix 96 × 78 pixels; frequency 60 Hz). Additional acquisition parameters are listed in Table S1 in the Supplemental Material. For the first part of the imaging protocol, the AC driver was placed in a standardized manner, as recommended by the manufacturer, over the right hepatic lobe using the xiphoid process and the right midclavicular line as anatomical landmarks. A fixed frequency of 60 Hz was transmitted through the upper abdomen. After completion of the protocol using the AC driver, the GT transducer was positioned at a similar position as the AC driver parallel to the right midclavicular line. However, its position was in the mid‐axillary line to align the transducer's main vibrational direction with the image acquisition's readout direction (i.e., in the right–left direction) to minimize possible motion‐induced artifacts. Both GT 2D/3D‐MRE acquisitions used the Ristretto scheme with fractional motion encoding, which has been described elsewhere.^23^ Note, that GT‐MRE has been acquired with a GRE sequence, whereas AC‐MRE used a SE‐EPI sequence (Table S1 in the Supplemental Material). Mechanical excitation frequency was maintained at 60 Hz for both acquisition schemes. Signals were acquired using the 18‐channel body matrix coil and (parts of) the table‐integrated 32‐channel spine matrix coil. At the end of each examination, study participants completed a satisfaction questionnaire (Fig. S1 in the Supplemental Material). Overall comfort included a range of factors that influence the patient's experience with the device, even when it is not active. This encompassed how well the device fit the patient's body, ensuring it feels secure and non‐restrictive. Additionally, we considered all aspects of the materials, including the surface properties of the transducer, as well as factors like portability, ease of use, and its aesthetic appeal. All items were rated on a 5‐point Likert scale, with 1 indicating “strongly agree” to 5 “strongly disagree.”

### Method for GT 2D‐ and 3D‐MRE Reconstruction of Stiffness

The 3D‐MRE reconstruction approach used in this study has previously been reported in detail.^22^, ^24^ In short, applying the mathematical curl‐operator allows to remove contributions of the compressional wave, which are—given the signal‐to‐noise ratio (SNR) in MRE data—not possible to be processed properly. The resulting Helmholtz equation allows for solving of the complex‐valued shear modulus $\left|G^{*}\right|$, i.e., for elasticity $G^{\prime }$ and viscosity $G^{\prime \prime }$ in a minimum $\chi^{2}$‐manner for the square of the wave‐vector ${\vec{k}}^{2}=\frac{\rho \omega^{2}}{G^{*}}$, where *ρ* is the tissue density (assumed to be constant and equal to that of water), and *ω* is the angular frequency of the mechanical vibration. However, here, only its magnitude $\left|G^{*}\right|=\sqrt{G\prime^{2}+G\prime \prime^{2}}$ is compared among different methods. For the GT‐2D reconstruction, the previously proposed directional filter approach has been used,^25^ which tries 1) to remove compressional wave contributions via high‐pass filters, 2) to simplify the inversion of the wave equation by selecting individual contributions from the entire displacement field in several angular directions by selecting a pie‐chart in Fourier space, and 3) to remove noise via low‐pass filters.

### Quality Indices for GT 2D‐ and 3D‐MRE Acquisitions

In the case of 2D‐MRE, it is impossible to rigorously calculate the displacement's curl‐field as it requires at least the measurement of two orthogonal motion components.^26^ Octahedral shear strain, which has been proposed as a possible SNR metric,^27^ requires unfortunately knowledge of all three spatial displacements. Hence, the amount of shear cannot be quantified unambiguously within a 2D‐MRE dataset. Therefore, our proposed 2D quality index (QI) utilizes only quantities linked to the measured displacement vector, i.e., on the one hand the local wave amplitude $A_{z}$, and on the other hand the quality of the temporal sinusoidal motion of a pixel (termed nonlinearity). It is quantified via a pixel‐wise Fourier transform and measures locally the pollution by noisy vibrational frequencies in percentage, i.e., a value of zero % indicates a perfect sinusoidal motion, whereas a value of for instance 50% indicates that 50% of the vibrational energy is not located within the main oscillation frequency but in other frequencies. The logarithm of this ratio, i.e., ${\mathrm{QI}}^{2D}=\log_{10}\left(\frac{A_{z}}{\text{nonlinearity}}\right)$ will be used as QI, with the logarithm allowing to easily incorporate scale changes as a DC shift of the cut‐off.

In case of 3D‐MRE, the magnitude of the curl $\left(\left|Q\right|=\left|\nabla \times \vec{u}\right|\right)$ can be quantified, as well as the magnitude of the divergence of the displacement $\left(\;|\mathrm{Div}|=|\nabla \vec{u}\;|=\left|\frac{∆V}{V}\right|=\left(1-2\sigma \right)\approx 0\right)$, which is very close to zero since tissue is incompressible (Poissons ratio σ=0.4999999).^28^ Thus, a pertinent metric to gauge the quality of the shear wave quantification is the ratio of the curl over the divergence, which should be equal to zero and thus estimates the noise, with values $\frac{\left|Q\right|}{\left|\mathrm{Div}\right|}>2$ indicative of good data. This quantity can be added to the previous QI^2D^ in a multiplicative fashion, i.e., ${\mathrm{QI}}^{3D}=\log_{10}\left(\frac{A_{\mathrm{tot}}}{\text{nonlinearity}}\frac{\left|Q\right|}{\left|\mathrm{Div}\right|}\right)$. To evaluate the pertinence of these QIs, we use superimposed analytic plane waves of known wavelength and attenuation. Knowledge of wavelength λ and attenuation α allows to calculate the corresponding shear modulus via $\left|G^{*}\right|=\left|\frac{\varrho \omega^{2}}{k^{2}}\right|,\;k=\frac{2\pi }{\lambda }+\mathrm{i\alpha}$. To create more complex wave patterns, individual plane waves are inclined randomly in 2D or 3D, respectively, and deteriorated with different amounts of noise for the 2D/3D reconstruction algorithms.

### Study Population

**FIGURE 1:**
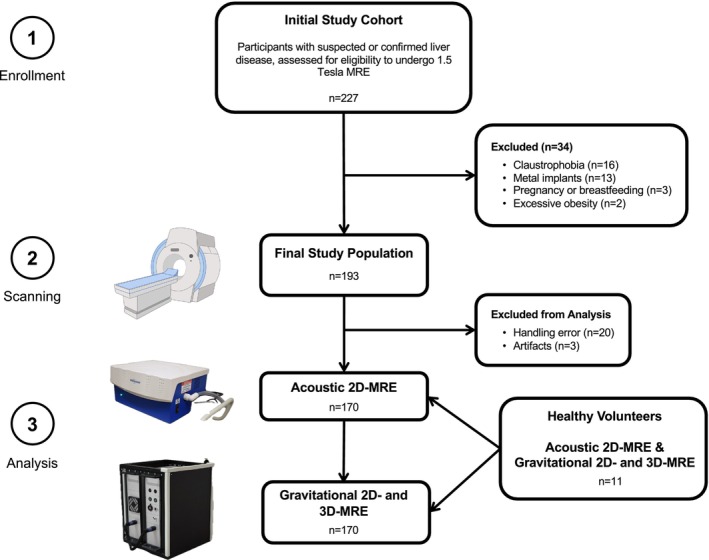
Study design. Out of an initial pool of 227 potentially eligible participants, 193 were ultimately recruited, and finally, 170 examinations were successfully recorded. In addition to these participants with suspected or confirmed liver disease, 11 healthy volunteers were investigated using the same AC‐ and GT imaging protocols. The most common reason for any technical failure was that the MRE system (AC) was in sleep mode and had not been turned on automatically, resulting in the absence of any mechanical waves in the data. AC = acoustic; GT = gravitational; MRE = magnetic resonance elastography.

In this prospective, cross‐sectional, single‐center study, participants older than 18 years with clinically suspected or confirmed liver disease were recruited at the University Hospital Frankfurt (Frankfurt am Main, Germany) between November 2022 and September 2023. As an expert center for fatty liver disease, a large portion of the study cohort consisted of patients with MASLD and MASH. Participants classified under MASLD did not exhibit any inflammatory changes in the microscopic slices of biopsy‐proven cases. Therefore, when we refer to the MASLD group, we are specifically referring to participants with MASLD without hepatitis. Exclusion criteria were the inability to undergo MRI (eg, claustrophobia, metallic implants, or obesity exceeding the bore size of the scanner [N = 31]), and pregnancy (N = 3). If available, pathological review had been conducted at the Institute of Pathology of the University Hospital Frankfurt am Main. Hepato‐pathologists with at least 6 years of experience assessed each biopsy to define the activity score and fibrosis stage according to the NASH Clinical Research Network (CRN) criteria in single reader sessions.^29^, ^30^, ^31^ All pathologies were classified based on either the histopathological result or the final adjudicated clinical diagnosis in cases where liver biopsy was not available. For participants without biopsy confirmation, a combination of clinical parameters and laboratory values was used to confirm liver disorders. In addition to patients with known or suspected liver disease, separate investigations were conducted on healthy volunteers without any known abdominal disease or suspicious findings in previously performed tests, as individually confirmed upon request. None of the healthy volunteers had undergone a liver biopsy, and a fibrosis stage of F0 could only be presumed. The study design is illustrated in Fig. 1.

### Hardware Setup

**FIGURE 2:**
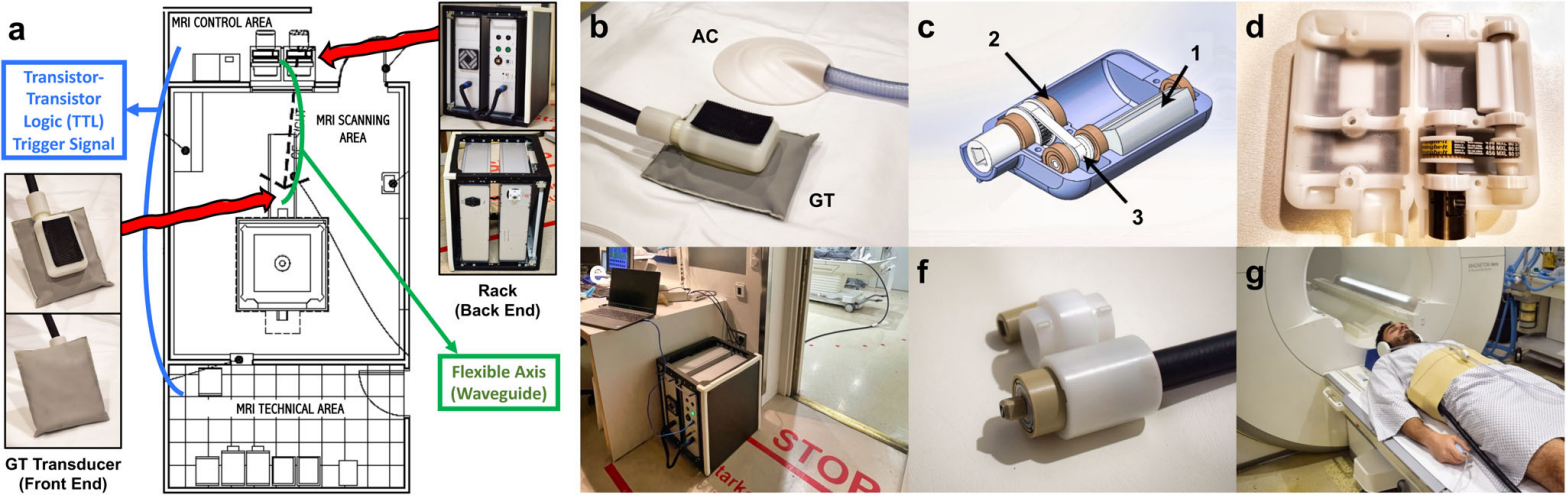
MRE hardware setup. (**a**) The research GT‐MRE system consists of an electronics rack and a stepper motor positioned outside the MRI scanning area (MRI control area). Connected to the MRI spectrometer via a trigger cable (blue line), it synchronizes with the MRE sequence on the spectrometer. A flexible rotating shaft extends through the waveguide into the scanner room until reaching the iso‐center of the magnet, where the GT transducer is connected (MRI scanning area). (**b**) Picture of the AC (above) and GT (below) transducer. (**c**) Schematic internal view of the GT transducer featuring the rotating mass (1), shaft mechanics (2), and gear belt (3). (**d**) View inside the GT transducer. (**e**) Perspective from the MRI control area. (**f**) Connection adapter between axis sections to reach 6 m total length. (**g**) Volunteer undergoing testing using the GT transducer. AC = acoustic; GT = gravitational; MRE = magnetic resonance elastography; TTL = transistor‐transistor logic trigger signal.

The GT‐MRE research demonstrator consisted of a rack located outside the MRI room housing electronics and motor, a flexible shaft connecting the motor with the GT transducer (two sections of 3 m each linked together via bayonet locknut connections) reaching through the waveguide into the MRI examination room, and the GT transducer itself, which was attached to the patient using a semi‐elastic belt (Fig. 2). The transducer was equipped with a curved contact plate to match the anatomy and a gel pad enclosed by a soft antibacterial cover to increase patient comfort (Fig. S2 in the Supplemental Material).

### Image Reconstruction and Evaluation

Confidence and stiffness maps for AC‐2D were created inline with the standard product inversion algorithm (“MMDI v3.0.4,” Resoundant, Rochester, MN, USA). Equivalent maps were created offline using our proposed research 2D/3D reconstruction algorithms, including corresponding maps of QIs. For the evaluation of outliers, GT‐3D has been used as the reference.

### Creation of Region of Interests (ROIs) for Elasticity Analysis

A radiologist with 5 years of experience in abdominal imaging (D.C.) and a biomedical engineer with 20 years of experience in MR elastography and 10 years of experience in liver imaging (R.S.) created ROIs for AC‐ and GT‐MRE on the corresponding anatomical images, blinded to clinical and histopathological information (Fig. S3 in the Supplemental Material). However, upon drawing ROIs, each reader was aware of which data she/he was treating, i.e., AC‐based or GT‐based data, due to the obvious difference in resolution of the corresponding MR‐magnitude images (in‐plane resolution for AC 1.5 × 1.5 mm^2^, and for GT 4 × 4 mm^2^). Both readers considered MRE confidence maps and liver anatomy before drawing ROIs to avoid extrahepatic anatomical structures. As shown in Fig. S3 in the Supplemental Material, ROIs aimed to cover the outer liver parenchyma by carefully avoiding large vessels from deeper liver regions. However, ROIs were drawn oblivious to any details of the wave propagation, such as plane‐wave behavior. While two independent readers set and assessed the ROIs, four radiologists and one biomedical engineer with at least 3 years of experience in abdominal imaging were involved in evaluating all MRE data sets.

### Statistical Analysis

Statistical analysis was conducted using MedCalc software (MedCalc Software Ltd., Version 22.016, Ostend, Belgium). Data normality was assessed utilizing Kolmogorov–Smirnov tests. Categorical variables were presented as numbers (percentages), whereas continuous variables were expressed as mean ± standard deviation (SD) for normally distributed data and as median with interquartile range (IQR) for non‐normally distributed data. For baseline differences between two groups, unpaired Student's *t* tests or Mann–Whitney *U* tests were used, as appropriate. The correlation between two ordinal variables was evaluated using Spearman's correlation coefficient (*r*). Comparisons of multiple measurements were performed using either the Wilcoxon matched‐pairs signed rank test or the Kruskal–Wallis test for multiple groups, with Dunn's posthoc test and Holm‐Bonferroni *P*‐value adjustment as needed. *P*‐values <0.05 were considered statistically significant.

Receiver operating characteristic (ROC) curve analyses were used to compare the performance of AC‐ and GT‐MRE in detecting liver fibrosis. For each ROC analysis, the area under the ROC curve (AUC), sensitivity, specificity, positive and negative predictive values were calculated.

Interreader reproducibility was assessed using Bland–Altman bias and reproducibility coefficients. Limits of agreement were defined as mean difference ±1.96 [SD]. Interreader agreement was evaluated using weighted *κ* statistics.^32^ A *κ*‐value of 0 indicated poor agreement, 0.01–0.20 slight agreement, 0.21–0.40 fair agreement, 0.41–0.60 moderate agreement, 0.61–0.80 substantial agreement, and a value of 0.81–1.00 indicated almost perfect agreement.

## Results

Out of the initial 193 participants (median age, 57 years [IQR, 47–65]; 78 females) with a median BMI of 27 kg/m^2^ (IQR, 24–31) who underwent MRE, 23 participants were ultimately excluded due to motion artifacts (three participants) or handling errors (20 participants), consisting of 1) improper coil placement (N = 3), 2) examination with a turned‐off GT device (N = 1), and 3) an AC device remaining in automatic sleep mode (N = 16). Of the remaining 170 participants (median age, 57 years [IQR, 46–65]; 66 females), 78 had a final diagnosis of MASH. Of those, 46 cases were biopsy‐proven, histologically classified into fibrosis stage F1 (N = 16), F2 (N = 10), F3 (N = 10), and F4 (N = 10). MASLD has been diagnosed in 50 participants with 13 biopsy‐proven cases, classified as stage F1 (N = 4), stage F2 (N = 4), stage F3 (N = 2), and stage F4 (N = 3). Additionally, 11 adult volunteers (median age, 31 years [IQR, 27–34]; 5 females) in healthy conditions without known abdominal disorders were presumed to have fibrosis stage F0 (BMI of 25 kg/m^2^ [IQR, 23–26]). Baseline characteristics of participants are presented in Table 1. The MRE protocol was completed within approximately 2.4 minutes using the GT transducer, like the commercial AC‐MRE solution (median of 2.4 minutes [IQR 2.4–2.5] vs. 2.5 minutes [IQR 2.5–2.6]; *P* = 0.16).

**TABLE 1:** Study Population

Variable—N (%), Mean ± SD or Median (IQR)	Overall (N = 170; 100%)	MASH (N = 78; 46%)	MASLD (N = 50; 29%)	Other Liver Disease (N = 42; 25%)	*P* Value
Demographic characteristics					
Age—year	55 ± 14	54 ± 14	57 ± 15	55 ± 15	0.553
Sex—no. (%)					
Male sex	104 (61%)	49 (63%)	32 (64%)	23 (55%)	
Female sex	66 (39%)	29 (37%)	18 (36%)	19 (45%)	
Race—no. (%)					
White	153 (90%)	69 (88%)	45 (90%)	39 (93%)	
Hispanic	4 (2%)	1 (1%)	1 (2%)	2 (5%)	
Black	5 (3%)	4 (5%)	‐	1 (2%)	
Asian	8 (5%)	4 (5%)	4 (8%)	‐	
BMI (kg/m^2^)					
All participants	27 (24–31)	28 (26–31)	26 (23–30)	25 (22–28)	0.561
Male	27 (24–31)	28 (27–31)	26 (24–30)	25 (22–29)	0.638
Female	27 (23–31)	29 (26–33)	25 (21–30)	25 (23–27)	0.869
Distribution—no. (%)					
<25	55 (32%)	13 (17%)	21 (42%)	21 (50%)	
25 to <30	67 (39%)	37 (47%)	16 (32%)	14 (33%)	
30 to <35	30 (18%)	16 (21%)	9 (18%)	5 (12%)	
≥35	18 (11%)	12 (15%)	4 (8%)	2 (5%)	
Etiology					
Fatty liver (final adjudicated clinical diagnosis)					
MASH—no. (%)	78 (46%)	78 (100%)	‐	‐	
MASLD—no. (%)	50 (29%)	‐	50 (100%)	‐	
Immune system abnormality					
Autoimmune hepatitis—no. (%)	2 (1%)	1 (1%)	1 (2%)	‐	
PSC—no. (%)	2 (1%)	1 (1%)	‐	1 (2%)	
PBC—no. (%)	2 (1%)	‐	1 (2%)	1 (2%)	
Drugs					
Drug induced liver injury—no. (%)	6 (4%)	3 (4%)	2 (4%)	1 (2%)	
Infection					
Hepatitis A—no. (%)	1 (1%)	‐	1 (2%)	‐	
Hepatitis B—no. (%)	11 (6%)	4 (5%)	5 (10%)	2 (5%)	
Hepatitis C—no. (%)	10 (6%)	2 (3%)	2 (4%)	6 (14%)	
Genetics					
Hemochromatosis—no. (%)	4 (2%)	1 (1%)	‐	3 (7%)	
M. Wilson—no. (%)	5 (3%)	2 (3%)	2 (4%)	1 (2%)	
Cancer and other growths					
Liver cancer—no. (%)	33 (19%)	6 (8%)	15 (30%)	12 (29%)	
Bile duct cancer—no. (%)	2 (1%)	‐	1 (2%)	1 (2%)	
Liver adenoma—no. (%)	13 (8%)	3 (4%)	7 (14%)	3 (7%)	
Other					
Alcohol abuse—no. (%)	28 (16%)	14 (18%)	5 (10%)	9 (21%)	
Sarcoidosis—no. (%)	3 (2%)	1 (1%)	1 (2%)	1 (2%)	
Non‐specific reactive hepatitis—no. (%)	3 (2%)	2 (3%)	‐	1 (2%)	
Complications					
Ascites	26 (15%)	6 (8%)	7 (14%)	13 (31%)	
Esophageal varices	26 (15%)	11 (14%)	5 (10%)	10 (24%)	
Gastrointestinal bleeding	20 (12%)	6 (8%)	6 (12%)	8 (19%)	
Portal hypertensive gastropathy	21 (12%)	7 (9%)	3 (6%)	11 (26%)	
Portal hypertension	31 (18%)	13 (17%)	6 (12%)	12 (29%)	
Hepatic encephalopathy	11 (6%)	3 (4%)	2 (4%)	6 (14%)	
Spontaneous bacterial peritonitis	4 (2%)	‐	1 (2%)	3 (7%)	
Hepatorenal syndrome	6 (4%)	4 (5%)	‐	2 (5%)	
Risk factors					
Type 2 diabetes	47 (28%)	29 (37%)	10 (20%)	8 (19%)	
Obesity	109 (64%)	64 (82%)	29 (58%)	16 (38%)	
Heavy alcohol use	30 (18%)	14 (18%)	5 (10%)	11 (26%)	
Family history of liver disease	10 (6%)	8 (10%)	2 (4%)	‐.‐	
Exposure to certain chemicals or toxins	3 (2%)	1 (1%)	1 (2%)	1 (2%)	
Type of care					
Outpatient	124 (73%)	68 (87%)	34 (68%)	22 (52%)	
Inpatient	46 (27%)	10 (13%)	16 (32%)	20 (48%)	
Mortality					
All‐cause death	4 (2%)	2 (1%)	1 (1%)	1 (1%)	

Baseline characteristics of participants with clinically suspected or confirmed liver disease at enrolment. AC = acoustic; BMI = body mass index; GT = gravitational; IQR = interquartile range; MASLD = metabolic dysfunction‐associated steatotic liver disease; MASH = metabolic dysfunction‐associated steatohepatitis; PBC = primary biliary cholangitis; PSC = primary sclerosing cholangitis; SD = standard deviation.

### Quality Indices

**FIGURE 3:**
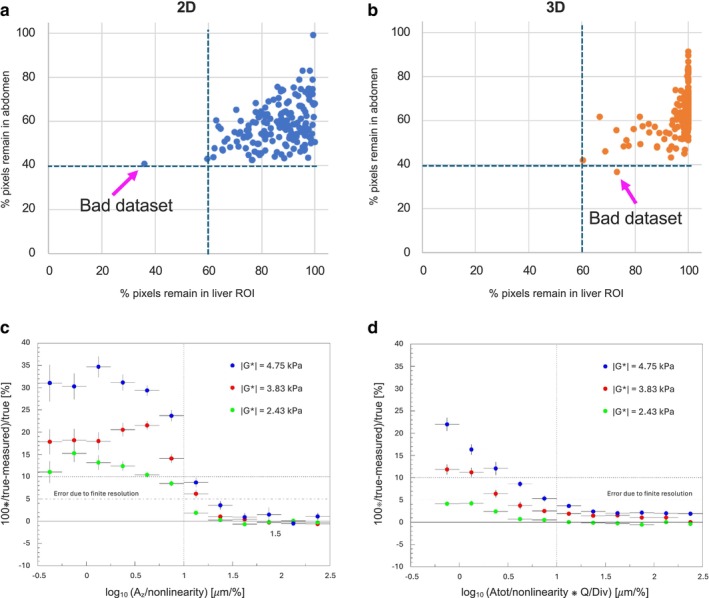
Performance of quality indices. (**a**, **b**) Percentage of remaining pixels within the liver ROI vs. percentage of remaining pixels within the entire abdomen, after application of the corresponding QIs for 2D and 3D, respectively. Meaningful cut‐off values are indicated by the dashed lines, whereby datasets exhibiting quality constraints can be spotted easily. Causes for quality constraints were typically missing vibrations (system not turned on) or a badly strapped transducer whereby strong motion artifacts were introduced, impacting nonlinearity and $\left|Q\right|/\left|\mathrm{Div}\right|$. (**c**, **d**) Percentage deviation of reconstructed stiffness $\left|G^{*}\right|$ as a function of corresponding QI for different true stiffnesses (2.43, 3.83, 4.75 kPa), for 2D and 3D, respectively. The proposed cut‐offs at 1 ensure a bias less than 10% for 2D and less than 5% for 3D. Mind that a given spatial resolution for the synthetic data of 4 mm (to match the in vivo resolution) does not always allow for reconstruction of the exact true wavelength due to clipping effects. The dotted line indicates the approximate limit given by the finite image resolution, which was assumed to be ~1/4 of a pixel. QI = quality index; ROI = region of interest.

Figure 3a,b show the percentage of pixels in GT‐MRE that remained valid within the entire abdomen for each patient vs. the corresponding number within the ROI after application of the QI for 2D and 3D, respectively. Datasets with quality constraints could easily be spotted by having abnormally few remaining pixels after QI. Figure 3c,d show the percentage deviation of reconstructed stiffness from the true value for 2D and 3D, respectively, as a function of the corresponding QI for synthetic data. A proposed cut‐off value of 1 ensures the validity of the result with a 10% deviation from the true value, as shown for true stiffnesses ranging from 2.4 to 4.8 kPa, thus covering the clinically relevant range of liver stiffnesses. The exact analytic solution is constrained by a finite image resolution of 4 mm in‐plane (2D) and additionally through‐plane (3D). As expected, stiffer objects with longer wavelengths require better SNR to recover unbiased stiffness values. For 3D, a cut‐off for ${\mathrm{QI}}_{3D}<1$ ensures a bias which is below the theoretical limit of ¼ of a pixel regarding the ability to resolve the wavelength and thus the stiffness correctly.

In patients, GT‐based MRE yielded similar exploitable areas (31.68 cm^2^ [IQR 24.21–40.01]) within the liver parenchyma as AC‐based MRE (29.84 cm^2^ [IQR 21.64–40.49]; *P* = 0.88) using their respective validity maps/QIs, hence meeting the quality control requirements. There was a small positive bias of 2.96% (95% CI: −1.99–7.90) for the covered area when using the GT approach, but it was not statistically significant (*P* = 0.24). A comparison of the valid surfaces of ROIs showed a good correlation between both methods (*R* ^2^ = 0.91).

### Correlation of AC/GT‐MRE With Histopathology

**FIGURE 4:**
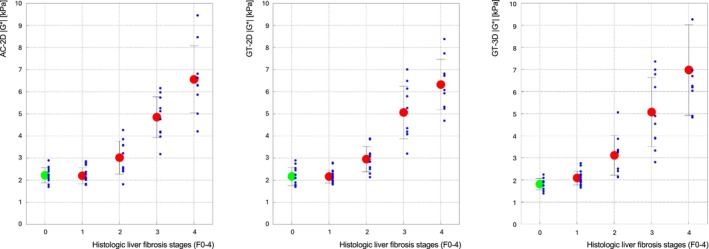
Stratification of stiffness according to histopathological liver fibrosis stages. Both AC‐ and GT‐MRE exhibited strong discriminatory ability across histopathological liver fibrosis stages (AUC ≥0.969 [95% CI: 0.865–0.998]), particularly within the clinically relevant range of F1–F3. Note that error bars indicate the variances for the different fibrosis grades, rather than the standard error of the mean. Red dots signify the mean of histologically confirmed cases, whereas the green dot represents the mean of all healthy volunteers included, classified as F0. AC = acoustic; AUC = area under the curve; GT = gravitational.

A strong significant positive relationship had been observed between histopathological fibrosis grading and AC‐2D (*r* = 0.87 [95% CI: 0.77–0.93]), GT‐2D (*r* = 0.88 [95% CI: 0.80–0.94]), and GT‐3D (*r* = 0.84 [95% CI: 0.72–0.91]). Both AC‐ and GT‐MRE demonstrated good discrimination of fibrosis stages (AUC ≥0.969 [95% CI: 0.865–0.998]), particularly within the clinically relevant range of F1–F3 (Fig. 4). GT‐3D showed a statistically significant difference between F0 and F1 in terms of stiffness (1.90 kPa [IQR 1.60–1.95] vs. 1.99 kPa [IQR 1.89–2.26]), whereas both 2D‐based approaches did not manage to reveal these subtle changes in biomechanics (Fig. 4c vs. Fig. 4a,b).

### Comparison Between AC‐2D and GT‐2D

**FIGURE 5:**
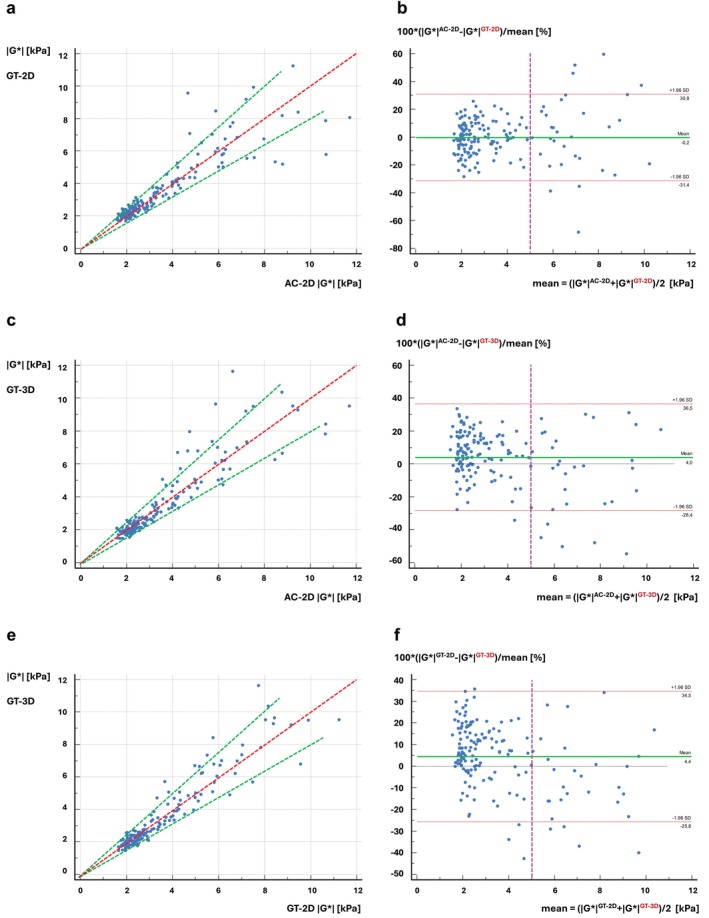
Comparison of AC 2D with GT 2D/3D‐MRE. Overall, the stiffness scatter plots show a high correlation between all three methods. (**a**, **b**) In the case of 2D, no systematic bias is noticeable between AC‐2D and GT‐2D, and significant differences only occur for isolated cases with mean stiffnesses >5 kPa, i.e., for very stiff cirrhotic livers where the difference is not clinically relevant. The dashed green lines indicate the ±20% deviation from identity (red dashed line). (**c**, **d**) The same holds for the comparison between AC‐2D and GT‐3D, although the 3D shows a systematic bias with AC‐2D overestimating values. Again, significant differences in stiffness occur only for mean stiffnesses >5 kPa, similar to the 2D case. The dashed green lines indicate the ±20% deviation from identity (red dashed line). (**e**, **f**) The correspondence between GT‐2D and GT‐3D was excellent, with a mean difference of $\bar{d}$=0.04%. AC = acoustic; GT = gravitational; MRE = magnetic resonance elastography.

AC‐2D and GT‐2D showed no significant differences in stiffness values (2.65 kPa [IQR 2.13–4.21] vs. 2.62 kPa [IQR 2.14–4.17]; *P* = 0.89) at high correlation (*r* = 0.89 [95% CI: 0.85–0.92]) (Table 2). The correspondence between both approaches was excellent, with a mean $\bar{d}$ of −0.23% (95% CI: −2.66–2.20; *P* = 0.85). Figure 5a,b show the scatter plot and relative Bland–Altman plot for the comparison between AC‐2D and GT‐2D, respectively.

**TABLE 2:** Histopathology

Variable	Total (N = 46)	F1 (N = 16)	F2 (N = 10)	F3 (N = 10)	F4 (N = 10)	*P* Value
MASH stiffness values—median (IQR)						
AC‐2D						
Stiffness (kPa)	2.65 (2.13–4.21)	2.10 (1.83–2.54)	2.90 (2.41–3.59)	4.86 (4.16–5.74)	6.31 (5.66–7.23)	<0.001
GT 2D‐MRE						
Stiffness (kPa)	2.62 (2.14–4.17)	2.04 (1.97–2.34)	2.96 (2.50–3.21)	4.81 (4.09–6.15)	6.08 (5.30–7.05)	<0.001
GT 3D‐MRE						
Stiffness (kPa)	2.47 (1.98–4.22)	1.99 (1.89–2.26)	3.26 (2.34–3.49)	4.72 (3.88–6.79)	6.26 (5.76–7.54)	<0.001
Final histopathological diagnosis—no.						
Definite MASH	46	16	10	10	10	‐
MASLD, not MASH	13	4	4	2	3	‐

Stiffness values of MASH patients stratified according to their histopathological grading. AC = acoustic; F = fibrosis stage; GT = gravitational; IQR = interquartile range; MASH = metabolic dysfunction‐associated steatohepatitis; MASLD = metabolic dysfunction‐associated steatotic liver disease; MRE = magnetic resonance elastography.

### Comparison Between AC‐2D and GT‐3D

Figure 5c,d show the scatter plot and the corresponding Bland–Altman plot for the comparison between AC‐2D and GT‐3D. The correlation between both methods was excellent (*r* = 0.91 [95% CI: 0.88–0.93]), however, showing a clear significant bias of +4.04% (95% CI: 1.45–6.63) with AC‐2D significantly overestimating stiffness values compared to GT‐3D (2.65 kPa [IQR 2.13–4.21] vs. 2.47 kPa [IQR 1.98–4.22]).

### Comparison Between GT‐2D and GT‐3D

Similarly to AC‐2D, GT‐2D demonstrated an excellent correlation with GT‐3D (*r* = 0.94 [95% CI: 0.92–0.96]), again with a significant overestimation by +4.37% (95% CI: 1.96–6.77) and significant difference in stiffness (2.62 kPa [IQR 2.14–4.17] vs. 2.47 kPa [IQR 1.98–4.22]) (Fig. 5e,f).

### AC/GT‐MRE Findings on Volunteers

The stiffness values of AC‐2D (2.20 kPa [IQR 2.00–2.48]) did not differ from GT‐2D (2.10 kPa [IQR 1.76–2.53]; *P* = 0.44) but did differ from GT‐3D (1.90 kPa [IQR 1.60–1.95]). The AC approach showed a substantial correlation with GT‐2D (*r* = 0.79 [95% CI: 0.35–0.94]) and a high correlation with GT‐3D (*r* = 0.86 [95% CI: 0.53–0.96]). GT 3D‐MRE allowed for a differentiation between F0 (1.90 kPa [IQR 1.60–1.95]) and F1 (1.99 kPa [IQR 1.89–2.26]), whereas AC‐ and GT 2D‐MRE showed no statistically significant difference (*P* ≥ 0.562) (Fig. 4).

### Diagnostic Performance of AC/GT‐MRE With Histopathology as Reference

The diagnostic performance of AC 2D‐MRE and GT 2D/3D‐MRE in discriminating between low‐grade and advanced fibrosis was excellent (AUC ≥0.969 [95% CI: 0.865–0.998]) (Table 3).

**TABLE 3:** Performance

Variable	Cut‐Off [kPa]	F1–F2 [kPa]	F3–F4 [kPa]	AUC	AUC	Sensitivity [%]	Specificity [%]	PPV [%]	NPV [%]
		(N = 27)	(N = 19)	(95% CI)	*P* Value	(95% CI)	(95% CI)	(95% CI)	(95% CI)
MASH stiffness values—median (IQR)									
AC‐2D									
Stiffness (kPa)	3.86	2.41 (2.03–2.78)	5.74 (4.35–6.30)	0.982 (0.892–1.000)	<0.001	96 (81–99)	95 (74–99)	96 (79–99)	95 (72–99)
GT 2D‐MRE									
Stiffness (kPa)	3.89	2.29 (1.99–2.79)	5.78 (4.44–6.41)	0.994 (0.912–1.000)	<0.001	100 (87–100)	95 (74–99)	96 (80–99)	100 (89–100)
GT 3D‐MRE									
Stiffness (kPa)	3.87	2.23 (1.94–2.71)	6.19 (4.62–6.94)	0.969 (0.865–0.998)	<0.001	96 (79–100)	90 (67–99)	92 (76–98)	94 (71–99)

Diagnostic performance of AC/GT‐MRE for discriminating between low‐grade and advanced liver fibrosis in histopathologically confirmed cases. AC = acoustic; AUC = area under the curve; F = fibrosis stage; GT = gravitational; IQR = interquartile range; MASH = metabolic dysfunction‐associated steatohepatitis; MRE = magnetic resonance elastography; NPV = negative predictive value; PPV = positive predictive value.

### Interreader Comparison

The interreader agreement for AC‐2D was high, with a mean $\bar{d}$ of −1.88% (95% CI: −4.17–0.42; *P* = 0.11) at a lower and upper limit of −14.36 (95% CI: −18.32–[−10.39]) and 10.61 (95% CI: 6.64–14.57), respectively, resulting in a coefficient of variation of 4.75% (95% CI: 3.52–5.99). The weighted *κ* was 0.91 (95% CI: 0.89–0.94).

The interreader agreement for GT‐2D was excellent, showing a mean $\bar{d}$ of 0.77% (95% CI: −2.27–3.80; *P* = 0.61). The lower and upper limits were −15.73 (95% CI: −20.97–[−10.49]) and 17.26 (95% CI: 12.02–22.50), respectively. The coefficient of variation was 6.07% (95% CI: 4.49–7.67), and the weighted *κ* 0.86 (95% CI: 0.81–0.91).

### Understanding the Outliers

**FIGURE 6:**
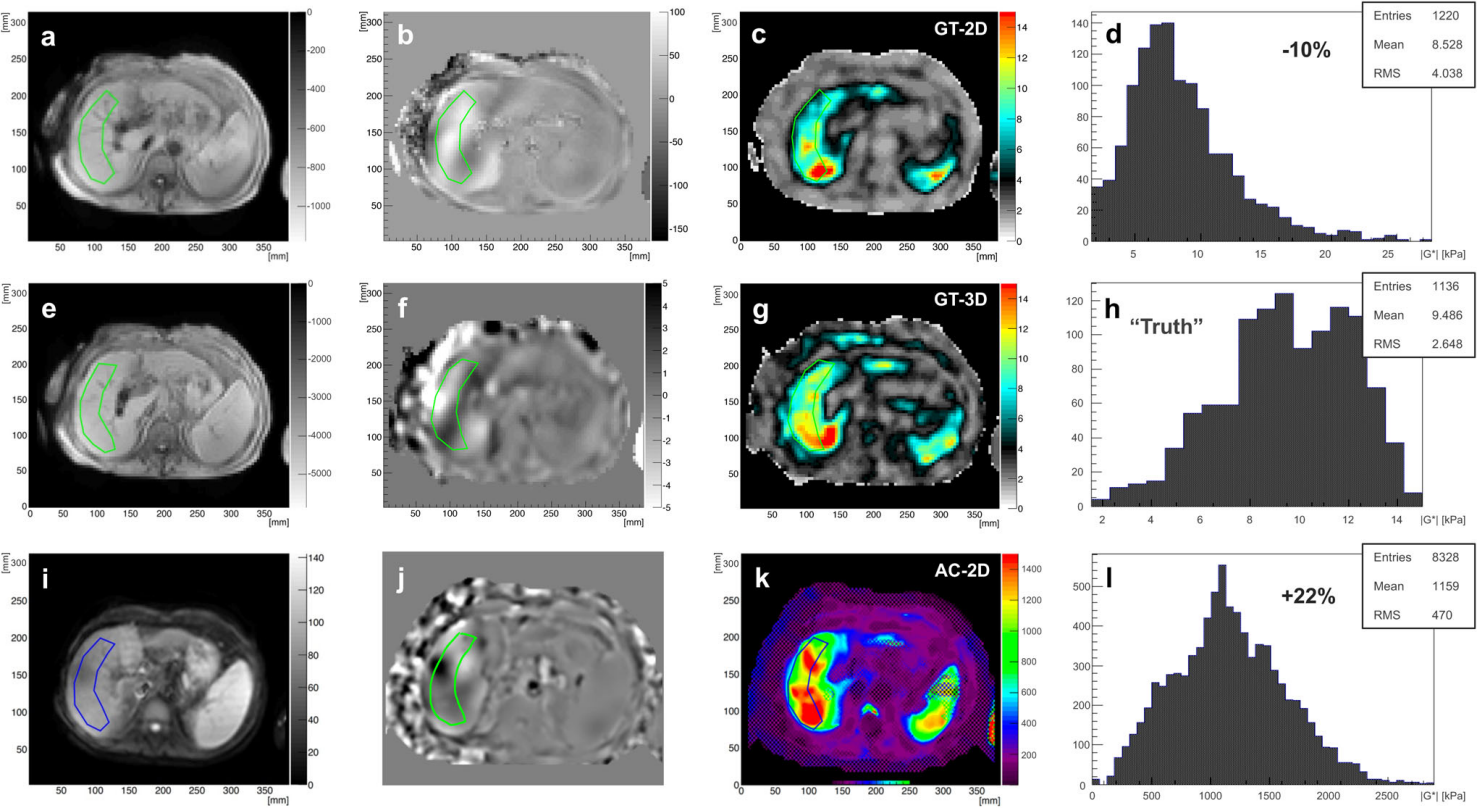
Outlier example. Stiffness estimates were compared among three different approaches: GT‐2D (**a–d**), GT‐3D (**e–h**), and AC‐2D (**i–**l), with MR magnitude, wave, stiffness, and distribution within ROI shown, respectively. Assuming that the 3D approach provides the least biased result, the GT‐2D underestimates the “true” stiffness by ~10%, whereas AC‐2D overestimates it by ~22%. Note that for figures (k) and (l), stiffness values are multiplied by 100. (b, f, j): Corresponding wave images for AC‐2D, GT‐2D, and GT‐3D MRE acquisitions. AC = acoustic; GT = gravitational.

An understanding of the outliers can be obtained using GT‐3D as a reference. Figure 6 shows a selected example from the outliers present in Fig. 5a. Here, AC‐MRE measures stiffness values significantly higher than the GT‐2D approach. Taking the stiffness estimate from the GT‐3D approach as a reference, it becomes obvious that the AC‐2D approach overestimates the true stiffness significantly by +22%, whereas the GT‐2D approach underestimates the true stiffness by −10%. Similar over‐/underestimation behavior has been observed for all outliers. Notably, all outlier cases occurred at mean stiffness values >5 kPa, where the exact value has no clinical relevance anymore since the patient is classified as F4.

### Feedback From Participants

The overall median scores for AC‐ and GT‐MRE were 1 (IQR 1–2) and 1.5 (IQR 1–2), respectively (*P* = 0.08). Overall comfort was the primary concern for participants following AC‐MRE (2, IQR 1–2), whereas for the GT system, it was the intensity of the vibrations (2, IQR 1–3) (Table S2 in the Supplemental Material).

## Discussion

Several MRI‐based techniques have been introduced as noninvasive alternatives to liver biopsy for the characterization of liver integrity,^33^ with biomechanics quantified via MRE currently proposing one of the most pertinent noninvasive imaging biomarkers.^34^ In our prospective evaluation of the GT 2D/3D‐MRE concept, we observed a strong correlation with AC‐2D and histopathological grading, along with an excellent agreement in Bland–Altman plots and among readers. Both methods effectively distinguished between low‐grade and advanced fibrosis. Proposed QIs facilitated the identification of pixels yielding a deviation above 10% from true stiffness values, based on a combination of total wave amplitude, temporal nonlinearity, and, additionally, SNR of the shear wave for 3D‐MRE.

The currently commercially available MRE medical device uses an AC approach to generate mechanical waves via a passive driver, bearing the risk of silencing due to preload (eg, tight positioning or patient weight) and the presence of upper harmonics.^17^, ^21^ Both factors may degrade wave quality and hinder accurate viscoelastic map reconstruction compared to mechanical systems.^17^, ^35^ To achieve a more accurate and reliable assessment of biomechanical tissue characteristics, the GT transducer concept has been introduced, which uses a spinning eccentric mass as a source for mechanical vibrations.^21^ GT‐MRE, in conjunction with a short echo time (TE) GRE sequence in this study, promises an alternative method for the noninvasive, accurate, and sensitive characterization of liver integrity.^21^

In our cross‐sectional prospective study involving 170 participants, GT 2D‐MRE agreed well with the current commercial solution (AC‐2D), showing high correspondence between both modalities. The interreader agreement for both 2D‐MRE methods was good. While the interreader variability for AC‐MRE appears to be lower—probably due to the higher spatial interpolation of the elasticity images leading to more subtle changes in the mean values when varying the shape of the ROI—the upper and lower limits of agreement overlap.

3D‐MRE data derived from the GT approach corresponded equally well with the GT 2D‐MRE and AC 2D‐MRE data. Both 2D‐MRE methods showed a clear significant bias with an overestimation compared to GT 3D‐MRE. This finding is similar to prior studies that have reported higher stiffness thresholds for 2D compared to 3D in the staging of advanced fibrosis and cirrhosis.^36^, ^37^ This discrepancy between 2D‐ and 3D‐MRE may originate from the specific way used to remove compressional wave components. For instance, in 2D, the resulting stiffness is minutely dependent on the chosen cut‐off value of the high‐pass filter.

The stiffness determined by both MRE approaches correlated well to histopathological fibrosis stages, corroborating findings from previous MRE studies in fatty liver disease.^38^, ^39^ Hepatic inflammation is believed to be a considerable contributor to increased liver stiffness, although the exact mechanism remains unclear.^18^ Alterations in the cellular volume and underlying inflammatory processes may impact shear wave propagation through liver parenchyma and consequently influence liver stiffness.^40^ With 3D‐MRE delivering further insight into subtle biomechanical alterations via quantification of, eg, phase‐angle and wave attenuation, it might allow for a more holistic assessment of liver biomechanics, potentially providing additional clinically relevant imaging biomarkers compared to the 2D approach that yields only $\left|G^{*}\right|.$ ^18^

The proposed QIs for GT‐2D and GT‐3D facilitated the simple identification of a corrupted scan by considering the percentage of remaining pixels within the abdomen and the liver ROI after application of the corresponding QI, and the removal of pixels carrying unreliable stiffness estimates. Results indicate that the proposed cut‐offs for 2D/3D ensure estimates for shear stiffness within 10% validity, particularly for the clinically relevant range up to approximately 5 kPa.

For AC‐MRE, overall comfort has been identified as the primary concern for the participants, whereas for GT‐MRE, it was the intensity of the vibrations. From our experiences, participants with low BMI complained the most about the strength of the vibrations. Consequently, two types of paddings (large/small gel pads) have been proposed to accommodate patients with different body types. This study has been consistently performed using a large gel pad. The discomfort experienced with AC‐MRE may stem from its flat membrane surface, which might not fully adapt to the curved abdominal wall.

### Limitations

First, the prospective enrollment of a large portion of participants with fatty liver disease resulted in an uneven distribution across the study groups. Second, the sample size of biopsy‐proven cases was limited due to strictly predefined criteria excluding patients with contraindications to undergo MRI scanning. Third, the use of liver biopsy as the reference standard may introduce the possibility of sampling error and interobserver variability. This highlights the superiority of MRE as an upcoming alternative, which enables a comprehensive evaluation of the entire liver. Fourth, our study warrants further multicenter validation involving more participants and ethnicities to assess inter‐center reproducibility.

## Conclusion

In conclusion, the tested GT‐MRE system demonstrated a strong correlation with the commercial medical product solution and provided comparable diagnostic value for liver stiffness assessment. Furthermore, the 3D‐MRE acquisition allows for the discrimination of healthy liver from low‐grade fibrosis and demonstrates excellent correlation to AC and GT 2D‐MRE. The proposed QIs can simplify the identification of corrupt datasets and ensure reliable stiffness estimates.

## Supporting information


**Figure S1:** Questionnaire. Illustration of the 5‐point Likert scale as part of the questionnaire analysis covering items such as “comfort,” “vibration,” “pain,” and “tightness.”

**Figure S2:** Transducer setup. The GT transducer was fitted with a curved contact plate and a gel pad, enclosed by a soft antibacterial cover, ensuring ergonomic and comfortable contact with the patient's abdomen (A, C). In contrast, the AC transducer featured a flat membrane surface, potentially limiting its ability to fully adapt to the curved abdominal wall (B, D). AC = acoustic; GT = gravitational.

**Figure S3:** ROI placement and histogram analysis. (A) Magnitude image of the 2D GRE‐MRE sequence showing the anatomy and the placement of the ROI (green polygon). (B) Wave image. (C, E) Corresponding image of the shear stiffness $\left|G^{*}\right|$ (in units of [kPa]) and stiffness distribution within the ROI, providing a mean of ~7 kPa. (D) Quality map. (F) Corresponding magnitude image of the 2D SE‐EPI commercial sequence showing the ROI placement (blue polygon). (G) Wave image. (H, J) Corresponding image of the shear stiffness $100∙\left|G^{*}\right|$ (in units of [kPa]) and stiffness distribution within the ROI yielding a mean of ~6.5 kPa. (i) Image of the shear stiffness with shadowing of pixels not meeting our quality criteria. EPI = echo‐planar‐imaging; GRE = gradient‐echo sequence; MRE = magnetic resonance elastography; ROI = region of interest; SE = spin‐echo.

**Table S1:** Study protocol. Detailed protocol of AC 2D‐MRE and GT 2D/3D‐MRE.

**Table S2:** Questionnaire ratings. Questionnaire ratings on the examination experience of AC‐ and GT‐MRE.
