# Supplementary material for: Biomechanical Assessment of Liver Integrity: Prospective Evaluation of Mechanical Versus Acoustic MR Elastography
Source: J Magn Reson Imaging. 2024 Aug 21;61(4):1890–904. doi: 10.1002/jmri.29560 (PMC11896941; doi:10.1002/jmri.29560)
Supplement: Supplementary file 1 — Figure S1: Questionnaire. Illustration of the 5‐point Likert scale as part of the questionnaire analysis covering items such as “comfort,” “vibration,” “pain,” and “tightness.” [file JMRI-61-1890-s004.pdf]

Questionnaire on MR-Elastography

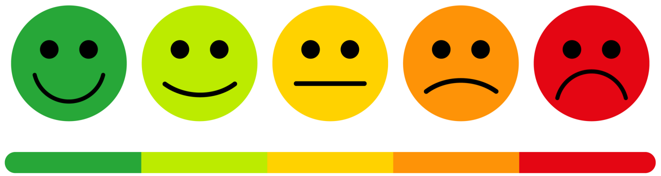

1	2	3	4	5
☐	☐	☐	☐	☐

Name and Birthday ________________

Frankfurt, Study Date ________________

Please rate the following items on a scale from 1 to 5:

Item	1	2	3	4	5	Item	1	2	3	4	5
1. Device (Acoustic): Comfort	☐	☐	☐	☐	☐	2. Device (Gravitational): Comfort	☐	☐	☐	☐	☐
1. Device (Acoustic): Vibration	☐	☐	☐	☐	☐	2. Device (Gravitational): Vibration	☐	☐	☐	☐	☐
1. Device (Acoustic): Pain	☐	☐	☐	☐	☐	2. Device (Gravitational): Pain	☐	☐	☐	☐	☐
1. Device (Acoustic): Tightness	☐	☐	☐	☐	☐	2. Device (Gravitational): Tightness	☐	☐	☐	☐	☐

Item	1	2	3	4	5	Item	1	2	3	4	5
Sound Intensity	☐	☐	☐	☐	☐	Care for the Participant	☐	☐	☐	☐	☐
Duration of the Examination	☐	☐	☐	☐	☐	Professionalism	☐	☐	☐	☐	☐
Shortness of Breath	☐	☐	☐	☐	☐	Study Information	☐	☐	☐	☐	☐
Comprehensibility	☐	☐	☐	☐	☐	General Satisfaction	☐	☐	☐	☐	☐
Physical Fatigue	☐	☐	☐	☐	☐	Length of Breathing Maneuvers	☐	☐	☐	☐	☐
