# Supplementary figures and images for: Biomechanical Assessment of Liver Integrity: Prospective Evaluation of Mechanical Versus Acoustic MR Elastography
Source: J Magn Reson Imaging. 2024 Aug 21;61(4):1890–904. doi: 10.1002/jmri.29560 (PMC11896941; doi:10.1002/jmri.29560)

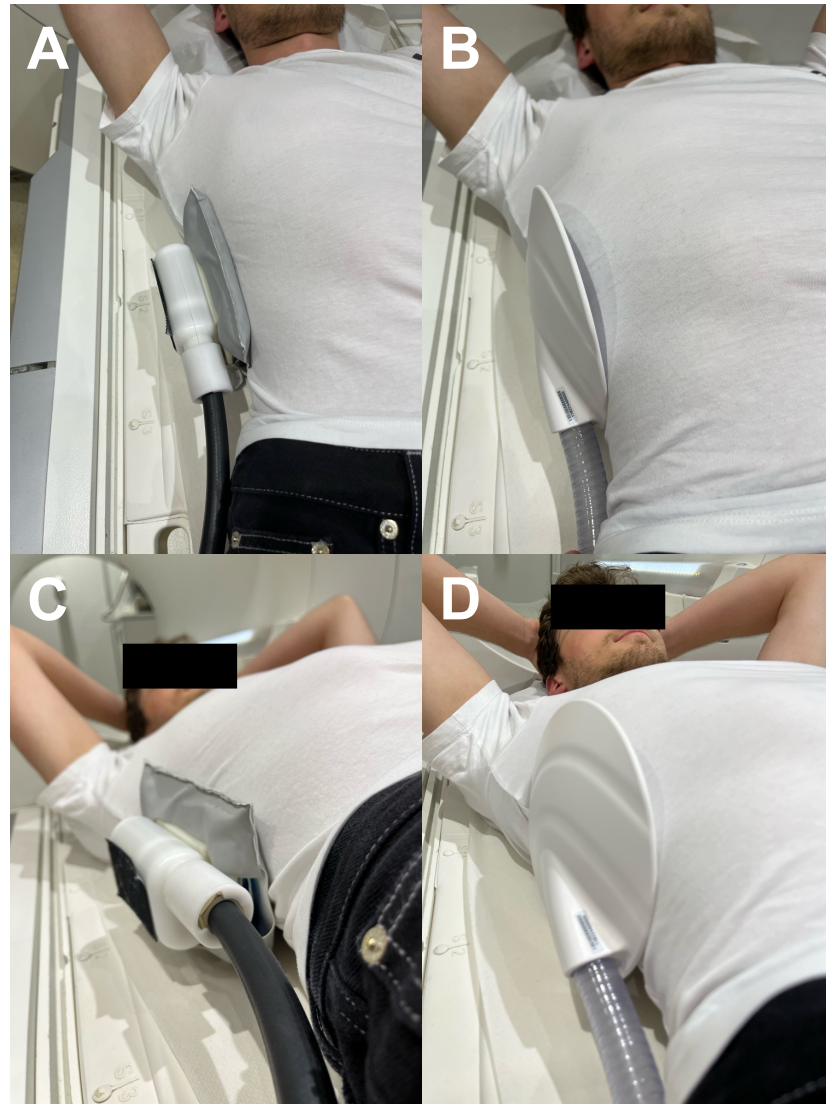

Supplement: Supplementary file 2 — Figure S2: Transducer setup. The GT transducer was fitted with a curved contact plate and a gel pad, enclosed by a soft antibacterial cover, ensuring ergonomic and comfortable contact with the patient's abdomen (A, C). In contrast, the AC transducer featured a flat membrane surface, potentially limiting its ability to fully adapt to the curved abdominal wall (B, D). AC = acoustic; GT = gravitational. [file JMRI-61-1890-s002.pdf]

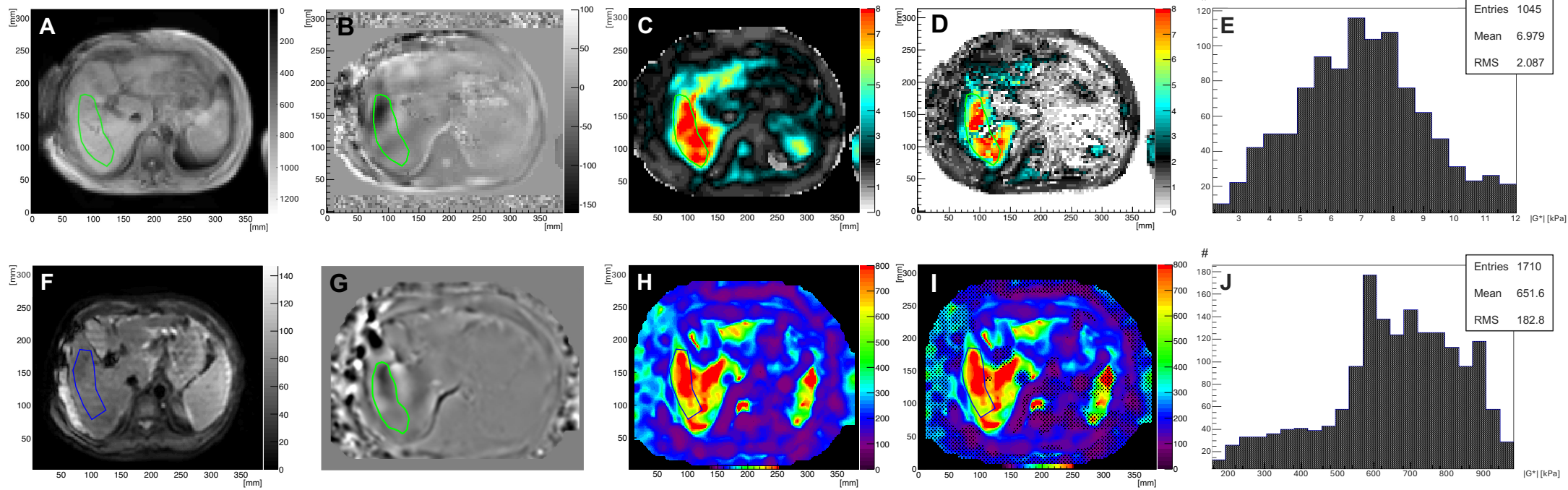

Supplement: Supplementary file 3 — Figure S3: ROI placement and histogram analysis. (A) Magnitude image of the 2D GRE‐MRE sequence showing the anatomy and the placement of the ROI (green polygon). (B) Wave image. (C, E) Corresponding image of the shear stiffness G* (in units of [kPa]) and stiffness distribution within the ROI, providing a mean of ~7 kPa. (D) Quality map. (F) Corresponding magnitude image of the 2D SE‐EPI commercial sequence showing the ROI placement (blue polygon). (G) Wave image. (H, J) Corresponding image of the shear stiffness 100∙G* (in units of [kPa]) and stiffness distribution within the ROI yielding a mean of ~6.5 kPa. (i) Image of the shear stiffness with shadowing of pixels not meeting our quality criteria. EPI = echo‐planar‐imaging; GRE = gradient‐echo sequence; MRE = magnetic resonance elastography; ROI = region of interest; SE = spin‐echo. [file JMRI-61-1890-s003.pdf]
