# Supplementary material for: Biomechanical Assessment of Liver Integrity: Prospective Evaluation of Mechanical Versus Acoustic MR Elastography
Source: J Magn Reson Imaging. 2024 Aug 21;61(4):1890–904. doi: 10.1002/jmri.29560 (PMC11896941; doi:10.1002/jmri.29560)
Supplement: Supplementary file 4 — Table S1: Study protocol. Detailed protocol of AC 2D‐MRE and GT 2D/3D‐MRE. [file JMRI-61-1890-s001.docx]

**Supplemental Table S1**

**Study Protocol |** Detailed protocol of AC 2D-MRE and GT 2D/3D-MRE.

|  | **AC 2D-MRE** | **GT 2D-MRE** | **GT 3D-MRE** |
| --- | --- | --- | --- |
| Sequence | Spin-echo EPI | Gradient-echo | Gradient-echo |
| Acquisition matrix (pixels) | 100x100 | 96x78 | 96x78 |
| Frequency (Hz) | 60 | 60 | 60 |
| TE (ms) | 46 | 9.53 | 9.53 |
| TR (ms) | 1000 | 54 | 104.25 |
| Flip angle (degree) | 90 | 25 | 25 |
| EPI-shots | Single shot | N/A | N/A |
| No. of breath holds (n), breath hold time (s) | 1, 11 | 1, 13 | 4, 14 |
| FOV (mm) | 380 | 384 | 384 |
| No. of slices (n), slice thickness (mm) | 5, 8 | 4, 8 | 8, 4 |
| Parallel imaging acceleration factor | 2 | 2 | 2 |
| Phase offsets | 4 | 4 | 4 |
| Receiver bandwidth (Hz/pixel) | 2084 | 400 | 400 |
| % FOV in phase-encoding direction | 100 | 81,3 | 81,3 |
| Motion encoding directions | S | S | M/P/S/Reference |
|  |  |  |  |

Abbreviations: AC, acoustic; EPI, echo planar imaging; FOV, field of view; GT, gravitational; MRE, magnetic resonance elastography; TE, echo time; TR, repetition time.
