# Supplementary material for: Biomechanical Assessment of Liver Integrity: Prospective Evaluation of Mechanical Versus Acoustic MR Elastography
Source: J Magn Reson Imaging. 2024 Aug 21;61(4):1890–904. doi: 10.1002/jmri.29560 (PMC11896941; doi:10.1002/jmri.29560)
Supplement: Supplementary file 5 — Table S2: Questionnaire ratings. Questionnaire ratings on the examination experience of AC‐ and GT‐MRE. [file JMRI-61-1890-s005.docx]

**Supplemental Table S2**

**Questionnaire Ratings |** Questionnaire ratings on the examination experience of AC- and GT-MRE.

| **Questionnaire Ratings**  **Likert Scale (1-5)**  **- mean (SD)** |  | **AC-MRE** | **GT-MRE** | P value  (T-test) | **Correlation Coefficient**  **(AC-MRE vs. GT-MRE)** | P value (Correlation) |
| --- | --- | --- | --- | --- | --- | --- |
|  |  |  |  |  |  |  |
| **Specific** |  |  |  |  |  |  |
|  |  |  |  |  |  |  |
| Comfort | - | 1.73 (0.78) | 1.70 (0.79) | 0.72 | 0.75 | <0.001 |
| Vibration | - | 1.71 (0.83) | 2.08 (0.99) | 0.01 | 0.62 | <0.001 |
| Pain | - | 1.35 (0.79) | 1.44 (0.80) | 0.32 | 0.66 | <0.001 |
| Tightness | - | 1.72 (0.81) | 1.74 (0.82) | 0.87 | 0.59 | <0.001 |
|  |  |  |  |  |  |  |
|  |  |  |  |  |  |  |
| **Common Experience** |  |  |  |  |  |  |
|  |  |  |  |  |  |  |
| Sound Intensity | 1.91 (0.95) | - | - | - | - | - |
| Length of Examination | 2.01 (0.95) | - | - | - | - | - |
| Shortness of Breath | 1.97 (1.10) | - | - | - | - | - |
| Comprehensibility | 1.19 (0.64) | - | - | - | - | - |
| Physical Fatigue | 1.56 (0.83) | - | - | - | - | - |
| Care for the Participant | 1.18 (0.58) | - | - | - | - | - |
| Professionalism | 1.21 (0.53) | - | - | - | - | - |
| Study Information | 1.32 (0.63) | - | - | - | - | - |
| General Satisfaction | 1.30 (0.68) | - | - | - | - | - |
| Length of Breathing Maneuvers | 2.24 (1.06) | - | - | - | - | - |
|  |  |  |  |  |  |  |

Abbreviations: AC, acoustic; GT, gravitational; IQR, interquartile range; MRE, magnetic resonance elastography; SD, standard deviation.
